# Association between blood pressure control status, visit-to-visit blood pressure variability, and cognitive function in elderly Chinese: A nationwide study

**DOI:** 10.3389/fpubh.2022.877192

**Published:** 2022-08-04

**Authors:** Luxinyi Xu, Ying Yang, Dan Cui

**Affiliations:** ^1^School of Public Health, Wuhan University, Wuhan, China; ^2^Global Health Institute, Wuhan University, Wuhan, China

**Keywords:** cognition, blood pressure, hypertension, control, antihypertension

## Abstract

**Background:**

Cognitive function is a concern among the elderly, which is related to the quality of life, life expectancy, and economic burdens. The relationship between blood pressure (BP) control status, visit-to-visit BP variability, and cognitive function remains controversial.

**Methods:**

We aimed to explore the association between BP control status at baseline, visit-to-visit BP variability, and cognitive function. This study included 3,511 elderlies in the China Health and Retirement Longitudinal Study, covering four waves for 7-year follow-up (baseline 2011, 2013, 2015, and 2018). BP was measured in Wave 2011, 2013, and 2015. Cognitive function was measured by Mini-Mental State Exam in Wave 2018. Participants were divided into two groups: mid-old group for reflecting midlife BP and cognition (45–59 years at baseline but aged 60 or over in Wave 2018), and old-old group for reflecting late-life BP and cognition (aged 60 or over at baseline). We use univariate analysis and general linear model to analyze.

**Results:**

Late-life BP showed stronger associations with cognitive function than midlife BP. As to late-life BP control status, controlled hypertension group get higher cognitive score than uncontrolled hypertension group in language (adjusted β = −0.34, 95%CI −0.68 to 0.00), and untreated hypertension group in orientation (adjusted β = −0.41, 95%CI −0.72 to −0.11), language (adjusted β = −0.35, 95%CI −0.67 to −0.04), and total (adjusted β = −0.99, 95%CI −1.85 to −0.12). Regarding visit-to-visit BP variability, midlife visit-to-visit systolic blood pressure (SBP) variability was associated with language (adjusted β = −3.70, 95% CI −5.83 to −1.57), while late-life visit-to-visit SBP variability was associated with orientation (adjusted β = −2.99, 95% CI −4.84 to −1.14), recall (adjusted β = −1.69, 95% CI −2.89 to −0.48), language (adjusted β = −2.26, 95% CI −4.13 to −0.38), and total (adjusted β = −9.50, 95% CI −14.71 to −4.28); Midlife diastolic blood pressure (DBP) variability and pulse pressure (PP) variability showed a significant relationship with language (adjusted β = 3.25, 95% CI −1.31 to −5.19) and calculation (adjusted β = −0.26, 95% CI −0.47 to −0.04), respectively. No significant correlation was found between midlife BP control status, late-life visit-to-visit DBP variability, late-life visit-to-visit PP variability, and cognitive score. There was no significant correlation between BP and memory.

**Conclusions:**

BP control status and visit-to-visit BP variability were significantly related to cognitive function among the Chinese elderly. Receiving effective late-life antihypertensive treatment and keeping SBP stable might contribute to prevent the development of cognitive impairment and dementia, especially for orientation and language function.

## Introduction

Dementia, an alarming problem among elderly patients, has reached 15.07 million and is predicted to rise to 22.2 million by 2030 in China (1). Dementia is characterized by a progressive decline in cognitive function until death, which will lower quality of life, shorten life expectancy, and even impose burdens on the family and society (2–4). Of note, it is estimated that the cost of treatment and care for the elderly with dementia is about 670 million to 900 million yuan annually in China (4). Paying attention to the cognitive function of the elderly is, therefore, an urgent problem for preventing mild cognitive impairment and dementia (5).

High blood pressure (BP) could cause cognitive decline *via* cerebral white matter lesions (6, 7). Central arterial stiffness increases with aging, leading to an increase in blood pressure as well as cerebral vascular damage (8, 9). Meanwhile, growing studies have confirmed the relationship between visit-to-visit BP variability and cognitive decline (7, 10, 11). It is found that per standard deviation increase in systolic blood pressure (SBP) and diastolic blood pressure (DBP), cerebral white matter lesions volume increased by 0.08 and 0.09 mL/y, respectively (7). Although the mechanism above has clearly demonstrated the relationship between aging, blood pressure, cognitive function, the evidence to support it by exploring the relationship between blood pressure and cognitive function in the follow-up population is still insufficient.

Progress has been made in proving the correlation between hypertension and cognitive impairment (12, 13), indicating that active antihypertensive treatment is of great significance to prevent cognitive impairment (14, 15), whereas there are still many controversies and deficiencies (16). Researchers focused on the association between midlife BP and late-life cognition, or late-life BP and late-life cognition. The majority of studies focused on the former, and found that midlife hypertension was an important risk factor for late-life cognitive impairment (14, 17, 18). For example, Gottesman et al. (15) also demonstrated that midlife hypertension, especially elevated SBP, was associated with decline in cognition, and emphasizing the importance of antihypertensive treatment in middle-age. In contrast, current studies on the relationship between late-life BP and cognition are inconsistent (14), some found that high late-life BP was risk factors for cognitive impairment (18, 19) while some indicated that there was no strong link between BP and dementia in older adults (18, 20, 21). In addition, previous studies have suggested that higher degree of visit-to-visit BP variability was related to higher risk of cognitive impairment and dementia (10, 20), indicating that further studies need to explore the relationship between BP variability and cognitive decline.

To date, previous studies mostly examined the influence of hypertension and antihypertensive treatment on cognitive impairment and dementia, rather than the quantitative relationship between blood pressure and cognitive function, ignoring that the decline in cognition is a progressive process (3, 22). Dementia patients tend to have a decline in the abilities of daily living, learning, work, and social communication (23). The Mini-Mental State Exam (MMSE) is the preferred scale for dementia screening (24, 25). Many studies have proved the high correlation between scores of each MMSE subitem and quality of life (26, 27), proving that subitem scores can provide more information than total scores, whereas studies always tend to focus only on the total MMSE score (20, 28–30). Thus, we aimed to analyze the relationship between BP and cognitive function of all dimensions to achieve detailed results.

The objectives of this study, was to explore the relationship between BP control status at baseline, visit-to-visit BP variability and cognitive scores in all cognitive dimensions in Chinese elderly for a 7-year follow-up, in order to contribute to provide some reference about BP controlling for preventing the development of cognitive impairment and dementia. We hypothesize that both variables were associated with cognition.

## Methods

### Data source

This study followed the STROBE Statement (31). We used data from the China Health and Retirement Longitudinal Study (CHARLS), a longitudinal survey conducted by National School of Development, Peking University. In the CHARLS, Chinese adults aged 45 years and older were investigated, and all samples were selected through a four-stage stratified multi-stage Precision Positioning System random sampling strategy, experiencing a step-by-step random selection of 28 provinces, 3 villages/communities, 150 counties, 80 households, 1 individual (32). The survey has been conducted since 2011 (baseline) and followed up every 2 or 3 years, up to now, three waves (2013, 2015, and 2018) national follow-up surveys have been conducted. In the four survey waves, 17,697 (Wave 2011), 18,254 (Wave 2013), 20,273 (Wave 2015) and 19,816 (Wave 2018) respondents were involved respectively, with respondent rates of 80.51, 82.63, 82.13, and 83.84% (33).

### Study population

**Figure 1:**
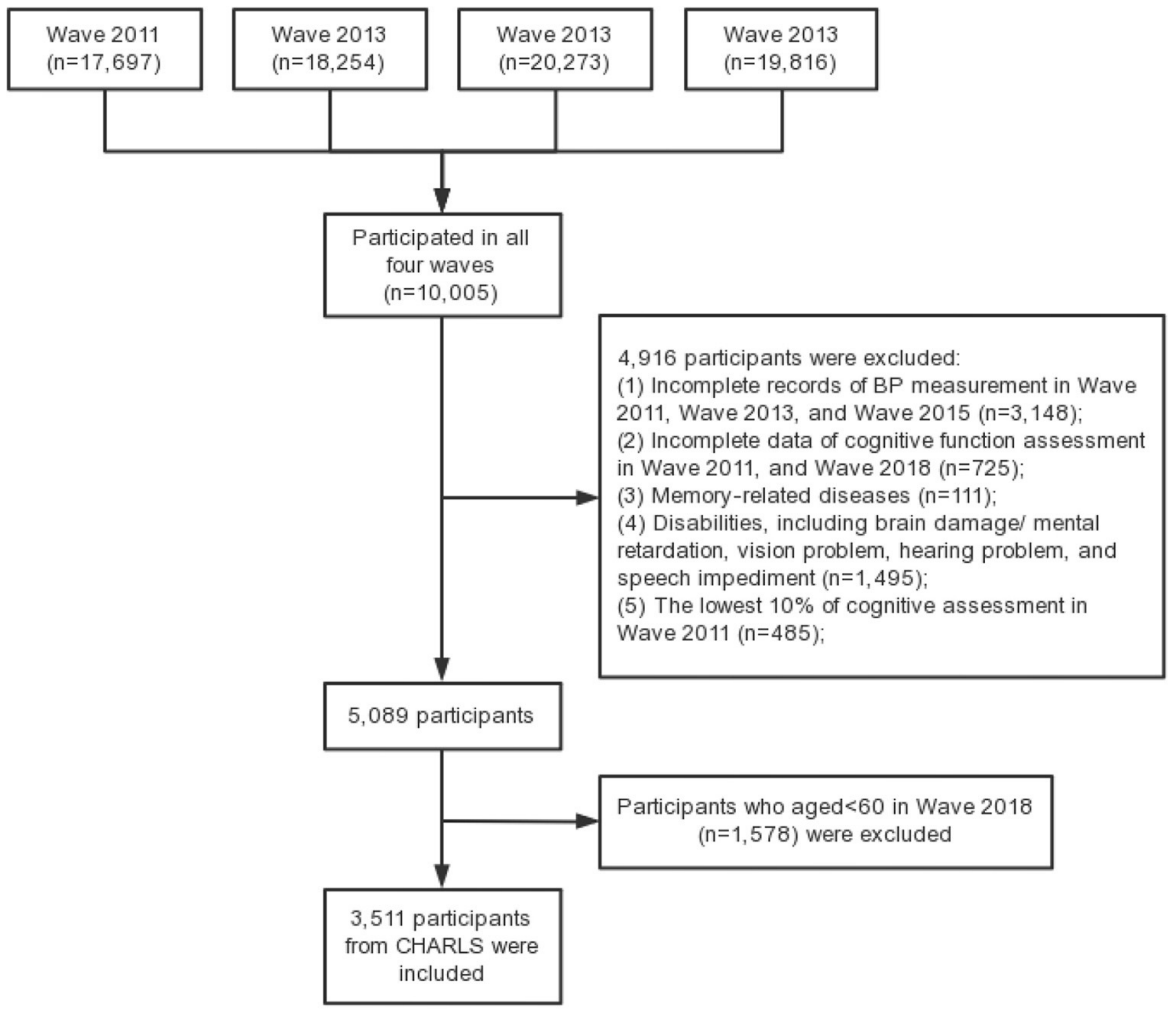
Flow chart for the sample selection.

In the present study, elderly people who participated in all four waves of CHARLS survey were included as study sample, with the following inclusion criteria: (a) aged 60 and over in Wave 2018; (b) complete records of BP measurement in Wave 2011, Wave 2013 and Wave 2015; (c) complete data of cognitive function assessment in Wave 2011 and Wave 2018. The exclusion criteria were set as follows: (a) cognitive impairment in Wave 1, that is, the lowest 10% of cognitive assessment, which is widely used to exclude participants with bad cognition (34–36); (b) memory-related diseases; (c) disabilities, including brain damage/mental retardation, vision problem, hearing problem, and speech impediment. Finally, 3,511 individuals were included in this study. Figure 1 shows the flow chart of the sample selection.

All individuals in this study were divided into two age groups (the mid-old and the old-old) based on their age at the baseline survey. According to the age classification of the World Health Organization (37), the mid-old group is defined as 45–59 years in Wave 2011 but aged 60 or over in Wave 2018, and the old-old group is defined as aged 60 or older in Wave 2011.

### Blood pressure measurement and classification

Data of BP was obtained from Wave 2011, 2013, and 2015. According to participants' ages of each wave, BP of the mid-old and the old-old was measured in midlife and late-life, reflecting midlife BP and late-life BP, respectively.

SBP and DBP were measured by trained investigators using an Omron HEM-7200 (38). Subjects who had smoked, exercised, eaten, or drunk within half an hour before measurement would be excluded. Investigators would put the cuff around the individual's left arm, place the arm on a flat surface with the palm facing up and the upper arm equal to the level of the heart. Each subject would be measured three times, and the average BP was used as the subject's final BP value.

### Exposure

Exposures included in this study covered two important aspects of midlife/late-life BP: (a) Midlife/late-life BP control status at baseline: according to “BP classification” and “antihypertensive treatment” in Wave 2011, there were four groups: controlled hypertension (normal BP with treatment), uncontrolled hypertension (high BP with treatment), untreated hypertension (high BP without treatment) and no hypertension (normal BP without treatment); (b) Midlife/late-life visit-to-visit BP variability was defined as the coefficient of variation (calculated as standard deviation divided by mean) (39) of three BP measurements (measured in Wave 2011, Wave 2013, and Wave 2015). Visit-to-visit systolic BP variability, diastolic BP variability, and pulse pressure (PP) variability were calculated. PP was equal to SBP minus DBP.

“BP classification” used BP measurement data in Wave 2011 and classified according to Guidelines for the Prevention and Treatment of Hypertension in China (Revised 2018) (40): “high BP” was defined as SBP ≥ 140 and/or DBP ≥ 90, and “normal BP” was defined as SBP <140 and DBP <90.

“Antihypertensive treatment” in Wave 2011 was obtained by asking “Are you now taking any of the following treatments to treat hypertension?” If the answer is “Chinese traditional medicine,” “western modern medicine,” or “other treatments”, the patient is considered to “with treatment”. If the answer is “none of the above”, the patient is considered to “without treatment”.

### Outcomes

The main outcomes in this study were cognitive function scores. Cognitive function was assessed by using MMSE in Wave 2018. MMSE was designed by Folstein et al. (41) in 1975 for rapid screening of cognitive impairment, and it is recommended for screening dementia by grade A with good reliability, validity, and internal consistency (24, 25). This scale consists of 11 items with 30 points, covering five dimensions including orientation (10 points), calculation (5 points), memory (3 points), recall (3 points), and language (9 points, including naming, repetition, 3-stage command, writing, and copying). The higher the score, the better the cognition. The assessment in Wave 2011 used simplified MMSE with 21 points, including orientation, calculation, memory, recall, and copying.

### Covariates

Covariates included age group (mid-old/old-old), gender (male/female), education (illiterate/literate), marital status (Married/divorced, widowed, or separated/unmarried), smoke (still have/quit/never), drink in the last year (more than once a month/less than once a month/never), sleeping time during the night (<6 h, 6– <7 h, 7– <8 h, 8– <9 h, and ≥9 h), body mass index (underweight, normal weight, overweight, and obese), diabetes or high blood sugar (yes/no), stroke (yes/no), dyslipidemia (yes/no), and antihypertensive treatment (yes/no). Body mass index (BMI) was calculated as weight in kilograms divided by height in meters squared, and was classified into four groups: <18.5 underweight, 18.5–23.9 normal weight, 24.0–27.9 overweight, and ≥28 obese (42). Diabetes or high blood sugar, stroke, dyslipidemia, and antihypertensive treatment were defined based on self-reported information of physicians by asking “Have you been diagnosed with…?” and “Are you now taking any of the following treatments to treat hypertension?” in Wave 2011.

### Statistical analyses

Continuous and classified variables were described by mean ± standard deviation ($\bar{x}$ ± s) and rate (%), respectively. Continuous variables were analyzed by *t*-test or analysis of variance, and classified variables were analyzed by chi-square test or Fisher's exact probability method. Multiple imputation was used to fill the missing values. The general linear model was used for regression analysis in this study. BP control status at baseline and visit-to-visit BP variability were set as explanatory variables of interest, and the cognitive score was set as dependent variables. All covariates were included as adjustment for modeling. Statistical analysis was performed using STATA 14.0 and SPSS 22.0. Charts were made using Excel 2016. *P*-value < 0.05 were considered statistically different.

## Results

### Baseline characteristics

Table 1 presents baseline characteristics of the study population in the mid-old group and old-old group. A total of 3,511 individuals were involved in this study, with an average age of 61.5 years. The overall samples were divided into two subgroups: mid-old group and old-old group, including 1,568 (44.7%) participants and 1,943 (55.3%) participants. There are significant differences in age, gender, marital status, smoking, sleeping time, BMI, and hypertension among the participants in each subgroup (all *p*-values < 0.05). Compared with the old-old group, SBP and PP of the mid-old group were lower while DBP was higher (all *p*-values < 0.05).

**Table 1:** Demographic characteristics of study population at baseline (Wave 2011).

**Variables**		**Total (*n* = 3,511)**	**Subgroup**		***t*-value/χ^2^**	***p*-value**
			**Mid-old (*n* = 1,568)**	**Old-old (*n* = 1,943)**		
Age ($\bar{x}$±s)		61.5 ± 6.3	56.1 ± 2.0	65.9 ± 5.1	2,602.50	0.001
Gender [*n* (%)]	Male	1,781 (50.7)	751 (21.4)	1,030 (29.3)	9.10	0.003
	Female	1730 (49.3)	817 (23.3)	913 (26.0)		
Education [*n* (%)]	Illiterate	905 (25.8)	405 (11.5)	500 (14.2)	0.004	0.950
	Literate	2,606 (74.2)	1,163 (33.1)	1,443 (41.1)		
Marital status [*n* (%)]	Married	3,110 (88.6)	1,466 (41.8)	1,644 (46.8)	69.50	<0.001
	Divorced/widowed/ separated	387 (11.0)	96 (2.7)	291 (8.3)		
	Unmarried	14 (0.4)	6 (0.2)	8 (0.2)		
Smoking [*n* (%)]	Still have	1,157 (33.0)	496 (14.1)	661 (18.8)	11.90	0.003
	Quit	320 (9.1)	120 (3.4)	200 (5.7)		
	Never	2,034 (57.9)	952 (27.1)	1,082 (30.8)		
Drinking in the past year [*n* (%)]*	More than once a month	888 (25.3)	392 (11.2)	496 (14.1)	0.40	0.820
	Less than once a month	239 (6.8)	111 (3.2)	128 (3.7)		
	Never	2,382 (67.9)	1,064 (30.3)	1,318 (37.6)		
Sleeping time during the night (h)*	<6	1,091 (31.2)	446 (12.8)	645 (18.5)	19.00	0.001
	6– <7	763 (21.8)	358 (10.2)	405 (11.6)		
	7– <8	694 (19.9)	335 (9.6)	359 (10.3)		
	8– <9	716 (20.5)	337 (9.6)	379 (10.8)		
	≥9	231 (6.6)	85 (2.4)	146 (4.2)		
BMI (kg/m^2^)*	Underweight	207 (5.9)	59 (1.7)	148 (4.3)	34.00	<0.001
	Normal weight	1,864 (53.5)	803 (23.1)	1,061 (30.5)		
	Overweight	1,032 (29.6)	498 (14.3)	534 (15.3)		
	Obese	380 (10.9)	191 (5.5)	189 (5.4)		
Diabetes or high blood sugar [*n* (%)]*	Yes	198 (5.7)	87 (2.5)	111 (3.2)	0.04	0.840
	No	3,285 (94.3)	1,467 (42.1)	1,818 (52.2)		
Stroke [*n* (%)]*	Yes	58 (1.7)	23 (0.7)	35 (1.0)	0.60	0.450
	No	3,442 (98.3)	1,536 (43.9)	1,906 (54.5)		
Dyslipidemia [*n* (%)]*	Yes	343 (9.9)	149 (4.3)	194 (5.6)	0.20	0.640
	No	3,119 (90.1)	1,396 (40.3)	1,723 (49.8)		
Hypertension [*n* (%)]*	Yes	870 (24.9)	334 (9.6)	536 (15.3)	18.20	<0.001
	No	2,626 (75.1)	1,226 (35.1)	1,400 (40.1)		
Antihypertensive treatment [*n* (%)]*	Yes	691 (77.2)	268 (29.9)	423 (47.3)	0.01	0.910
	No	204 (22.8)	80 (8.9)	124 (13.9)		
SBP [mmHg, ($\bar{x}$ ± s)]		130.3 ± 20.7	128.2 ± 19.8	132.1 ± 21.2	−5.60	<0.001
DBP [mmHg, ($\bar{x}$ ± s)]		75.1 ± 11.9	76.0 ± 12.3	74.4 ± 11.4	4.10	<0.001
PP [mmHg, ($\bar{x}$ ± s)]		55.2 ± 14.3	52.2 ± 12.3	57.7 ± 15.3	−11.60	<0.001

*Indicates that there were missing data in the variable.

*SBP, systolic blood pressure; DBP, diastolic blood pressure; PP, pulse pressure; BMI, body mass index*.

### BP status at baseline and cognitive scores

**Figure 2:**
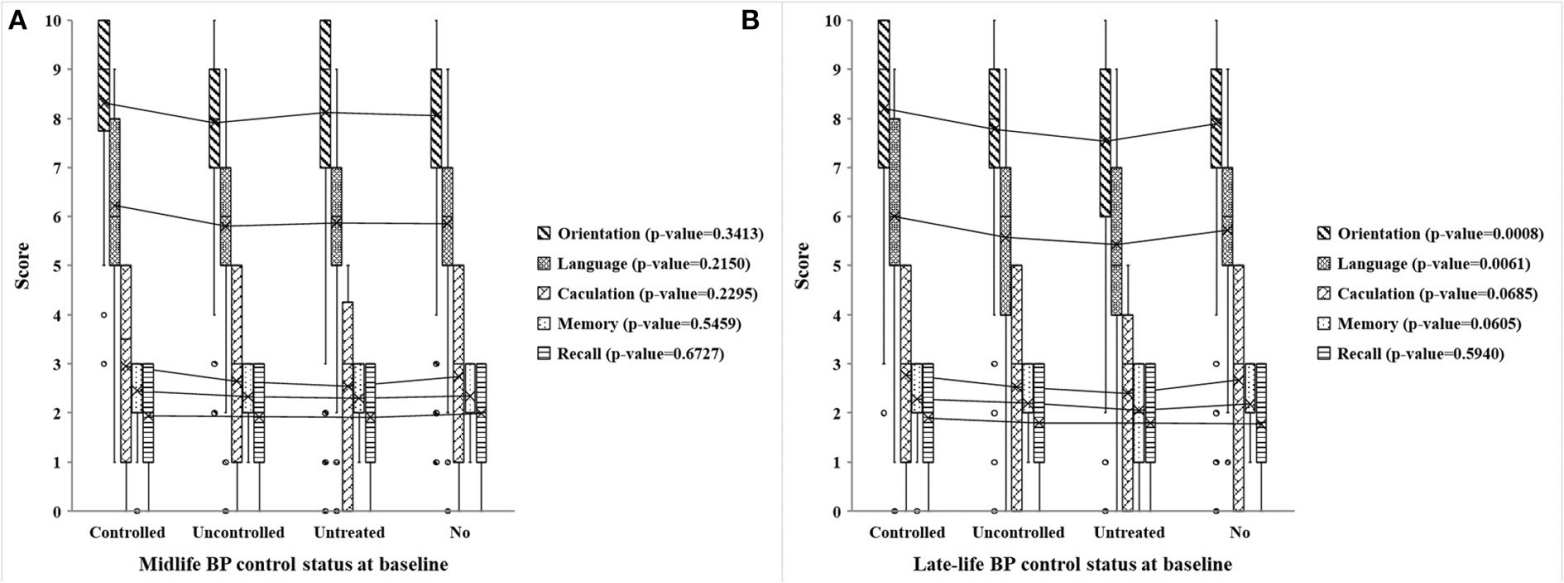
Scores of five cognitive dimensions in different midlife and late-life BP control statuses at baseline. **(A,B)** Respectively, show the cognitive scores of five dimensions (including orientation, language, calculation, memory, and recall) in different midlife and late-life BP control statuses at baseline. The midlife BP control status is present in the mid-old group, while the late-life BP control status is present in the old-old group. Controlled, uncontrolled, untreated, no present controlled hypertension, uncontrolled hypertension, untreated hypertension, and no hypertension, respectively. *P*-value is the analysis of variance of a certain cognitive dimension of four BP control statuses at baseline.

Figure 2 illustrates the distribution and univariate analysis results of cognitive scores among participants with different midlife and late-life BP control statuses. It is shown that four types of late-life BP control statuses differed significantly in cognitive scores of orientation, language, and total (all *p*-value < 0.001) (*P*-value of total score were shown in Supplementary Table S1), whereas no significant difference was observed in different midlife BP control statuses. Besides, referring to the orientation and language scores, the late-life controlled hypertension group had the highest scores while the late-life untreated hypertension group scored the lowest.

As shown in Tables 2, 3, midlife BP control status at baseline was related to cognitive scores of language, while late-life BP control status at baseline was associated with all cognitive dimensions except recall in crude analyses. However, after fully adjusting for demographic factors, the associations were attenuated. Especially, midlife BP control status was no longer with any cognitive dimensions.

**Table 2:** General linear model for association between BP control status at baseline, visit-to-visit BP variability and cognitive scores in the mid-old group.

**Model**	**Variables**	**Orientation**		**Memory**		**Calculation**		**Recall**		**Language**		**Total**	
		**β (95% CI)**	***p*-value**	**β (95% CI)**	***p*-value**	**β (95% CI)**	***p*-value**	**β (95% CI)**	***p*-value**	**β (95% CI)**	***p*-value**	**β (95% CI)**	***p*-value**
Crude model	**BP control status at baseline**												
	Controlled hypertension	Ref.		Ref.		Ref.		Ref.		Ref.		Ref.	
	Uncontrolled hypertension	−0.40 (−0.85, 0.06)	0.088	−0.11 (−0.34, 0.12)	0.332	−0.30 (−0.77, 0.17)	0.214	−0.02 (−0.28, 0.24)	0.875	−0.39 (−0.86, 0.07)	0.096	−1.22 (−2.60, 0.16)	0.083
	Untreated hypertension	−0.19 (−0.59, 0.22)	0.365	−0.15 (−0.35, 0.06)	0.155	−0.41 (−0.83, 0.01)	0.053	−0.03 (−0.26, 0.20)	0.807	−0.32 (−0.74, 0.09)	0.121	−1.10 (−2.32, 0.12)	0.078
	No hypertension	−0.28 (−0.63, 0.07)	0.120	−0.11 (−0.29, 0.06)	0.211	−0.22 (−0.58, 0.15)	0.242	0.04 (−0.17, 0.24)	0.736	−0.43 (−0.79, −0.07)	0.018	−1.00 (−2.07, 0.06)	0.064
	**Visit-to-visit BP variability**												
	SBP variability	−1.66 (−4.02, 0.69)	0.166	−0.52 (−1.71, 0.66)	0.387	−0.91 (−3.34, 1.52)	0.462	−1.06 (−2.41, 0.30)	0.126	−4.99 (−7.39, −2.59)	<0.001	−9.14 (−16.28, −2.00)	0.012
	DBP variability	1.30 (−0.85, 3.44)	0.237	0.46 (−0.62, 1.54)	0.406	0.47 (−1.74, 2.69)	0.676	0.52 (−0.71, 1.76)	0.407	4.14 (1.95, 6.33)	<0.001	6.88 (0.37, 13.40)	0.038
	PP variability	0.04 (−0.20, 0.28)	0.757	−0.04 (−0.16, 0.08)	0.497	−0.23 (−0.47, 0.02)	0.073	−0.04 (−0.18, 0.10)	0.56	−0.01 (−0.25, 0.24)	0.964	−0.28 (−1.00, 0.45)	0.455
Adjusted model	**BP control status at baseline**												
	Controlled hypertension	Ref.		Ref.		Ref.		Ref.		Ref.		Ref.	
	Uncontrolled hypertension	−0.25 (−0.65, 0.16)	0.229	−0.07 (−0.29, 0.15)	0.526	−0.16 (−0.58, 0.26)	0.461	0.01 (−0.24, 0.27)	0.931	−0.26 (−0.68, 0.15)	0.207	−0.73 (−1.87, 0.41)	0.212
	Untreated hypertension	0.05 (−0.32, 0.40)	0.808	−0.09 (−0.28, 0.11)	0.392	−0.19 (−0.57, 0.18)	0.312	0.03 (−0.20, 0.26)	0.793	−0.13 (−0.50, 0.24)	0.492	−0.33 (−1.36, 0.69)	0.524
	No hypertension	0.00 (−0.32, 0.33)	0.985	−0.04 (−0.21, 0.13)	0.651	−0.02 (−0.36, 0.32)	0.911	0.09 (−0.11, 0.30)	0.377	−0.23 (−0.56, 0.10)	0.173	−0.19 (−1.11, 0.72)	0.678
	**Visit-to-visit BP variability**												
	SBP variability	−0.24 (−2.32, 1.83)	0.817	−0.05 (−1.17, 1.07)	0.930	0.31 (−1.86, 2.48)	0.779	−0.65 (−1.97, 0.67)	0.335	−3.70 (−5.83, −1.57)	0.001	−4.33 (−10.24, 1.58)	0.151
	DBP variability	0.18 (−1.71, 2.07)	0.850	0.17 (−0.86, 1.19)	0.751	−0.48 (−2.46, 1.49)	0.633	0.26 (−0.94, 1.46)	0.675	3.25 (1.31, 5.19)	0.001	3.37 (−2.01, 8.75)	0.219
	PP variability	0.00 (−0.21, 0.21)	0.973	−0.06 (−0.17, 0.06)	0.341	−0.26 (−0.47, −0.04)	0.022	−0.05 (−0.18, 0.09)	0.490	−0.05 (−0.26, 0.16)	0.646	−0.40 (−1.00, 0.19)	0.183

*Adjusted Model was fully adjusted for age, gender, education, marital status, drink, smoke, BMI, sleeping time during the night, diabetes or high blood sugar, stroke, and dyslipidemia.

*Visit-to-visit SBP variability, DBP variability, and PP variability were set as continuous variables in the crude and adjusted models*.

*β, beta; Ref, reference; BP, blood pressure; SBP, systolic blood pressure; DBP, diastolic blood pressure; PP, pulse pressure*.

**Table 3:** General linear model for association between BP control status at baseline, visit-to-visit BP variability and cognitive scores in the old-old group.

**Model**	**Variables**	**Orientation**		**Memory**		**Calculation**		**Recall**		**Language**		**Total**	
		**β (95% CI)**	***p*-value**	**β (95% CI)**	***p*-value**	**β (95% CI)**	***p*-value**	**β (95% CI)**	***p*-value**	**β (95% CI)**	***p*-value**	**β (95% CI)**	***p*-value**
Crude model	**BP control status at baseline**												
	Controlled hypertension	Ref.		Ref.		Ref.		Ref.		Ref.		Ref.	
	Uncontrolled hypertension	−0.38 (−0.77, 0.01)	0.054	−0.08 (−0.28, 0.12)	0.448	−0.21 (−0.60, 0.17)	0.277	−0.08 (−0.31, 0.14)	0.461	−0.41 (−0.80, −0.02)	0.039	−1.16 (−2.34, 0.01)	0.052
	Untreated hypertension	−0.68 (−1.03, −0.33)	<0.001	−0.24 (−0.42, −0.06)	0.011	−0.39 (−0.73, −0.04)	0.029	−0.12 (−0.32, 0.09)	0.257	−0.58 (−0.93, −0.24)	0.001	−2.00 (−3.06, −0.94)	<0.001
	No hypertension	−0.31 (−0.62, 0.00)	0.051	−0.12 (−0.28, 0.05)	0.161	−0.13 (−0.43, 0.18)	0.423	−0.15 (−0.32, 0.04)	0.115	−0.29 (−0.60, 0.02)	0.063	−0.99 (−1.93, −0.05)	0.039
	**Visit-to-visit BP variability**												
	SBP variability	−4.08 (−6.23, −1.93)	<0.001	−1.14 (−2.27, −0.02)	0.046	−2.73 (−4.87, −0.60)	0.012	−1.92 (−3.16, −0.67)	0.003	−3.14 (−5.29, −0.99)	0.004	−13.01 (−19.53, −6.49)	<0.001
	DBP variability	1.27 (−0.77, 3.31)	0.222	0.11 (−0.95, 1.18)	0.835	1.62 (−0.41, 3.64)	0.117	0.81 (−0.37, 1.99)	0.180	0.96 (−1.08, 2.99)	0.357	4.77 (−1.41, 10.95)	0.131
	PP variability	0.03 (−0.15, 0.21)	0.742	0.02 (−0.08, 0.11)	0.723	−0.01 (−0.19, 0.17)	0.933	0.07 (−0.04, 0.17)	0.225	0.07 (−0.11, 0.25)	0.457	0.18 (−0.38, 0.73)	0.536
Adjusted model	**BP control status at baseline**												
	Controlled hypertension	Ref.		Ref.		Ref.		Ref.		Ref.		Ref.	
	Uncontrolled hypertension	−0.30 (−0.63, 0.04)	0.084	−0.03 (−0.23, 0.16)	0.734	−0.09 (−0.42, 0.24)	0.586	−0.05 (−0.27, 0.17)	0.662	−0.34 (−0.68, 0.00)	0.049	−0.81 (−1.76, 0.14)	0.093
	Untreated hypertension	−0.41 (−0.72, −0.11)	0.008	−0.12 (−0.30, 0.05)	0.170	−0.10 (−0.40, 0.21)	0.535	0.00 (−0.20, 0.20)	0.984	−0.35 (−0.67, −0.04)	0.026	−0.99 (−1.85, −0.12)	0.026
	No hypertension	−0.21 (−0.49, 0.06)	0.131	−0.08 (−0.24, 0.08)	0.319	−0.03 (−0.30, 0.24)	0.826	−0.10 (−0.28, 0.08)	0.293	−0.23 (−0.51, 0.05)	0.104	−0.65 (−1.43, 0.13)	0.101
	**Visit-to-visit BP variability**												
	SBP variability	−2.99 (−4.84, −1.14)	0.002	−0.84 (−1.90, 0.23)	0.123	−1.73 (−3.56, 0.10)	0.064	−1.69 (−2.89, −0.48)	0.006	−2.26 (−4.13, −0.38)	0.018	−9.50 (−14.71, −4.28)	<0.001
	DBP variability	0.52 (−1.23, 2.28)	0.559	0.04 (−0.97, 1.05)	0.934	0.93 (−0.81, 2.67)	0.293	0.80 (−0.34, 1.94)	0.170	0.59 (−1.19, 2.37)	0.514	2.89 (−2.07, 7.84)	0.253
	PP variability	0.05 (−0.11, 0.21)	0.535	0.02 (−0.07, 0.11)	0.706	0.02 (−0.14, 0.17)	0.846	0.07 (−0.04, 0.17)	0.199	0.07 (−0.09, 0.23)	0.391	0.22 (−0.23, 0.66)	0.333

*Adjusted Model was fully adjusted for age, gender, education, marital status, drink, smoke, BMI, sleeping time during the night, diabetes or high blood sugar, stroke, and dyslipidemia.

*Visit-to-visit SBP variability, DBP variability, and PP variability were set as continuous variables in the crude and adjusted models*.

*β, beta; Ref, reference; BP, blood pressure; SBP, systolic blood pressure; DBP, diastolic blood pressure; PP, pulse pressure*.

In summary, late-life BP status at baseline showed significant relationships with cognitive scores of orientation, language, and total in the adjusted model (all *p*-value < 0.05). Specifically, compared with controlled hypertension group, uncontrolled hypertension group performed worse in cognitive scores of language (adjusted β = −0.34, 95%CI −0.68 to 0.00, *p*-value = 0.049); untreated hypertension group performed worse in cognitive scores of orientation (adjusted β = −0.41, 95%CI −0.72 to −0.11, *p*-value = 0.008), language (adjusted β = −0.35, 95%CI −0.67 to −0.04, *p*-value = 0.0026), and total (adjusted β = −0.99, 95%CI −1.85 to −0.12, *p*-value = 0.0026).

### Visit-to-visit BP variability and cognitive scores

**Figure 3:**
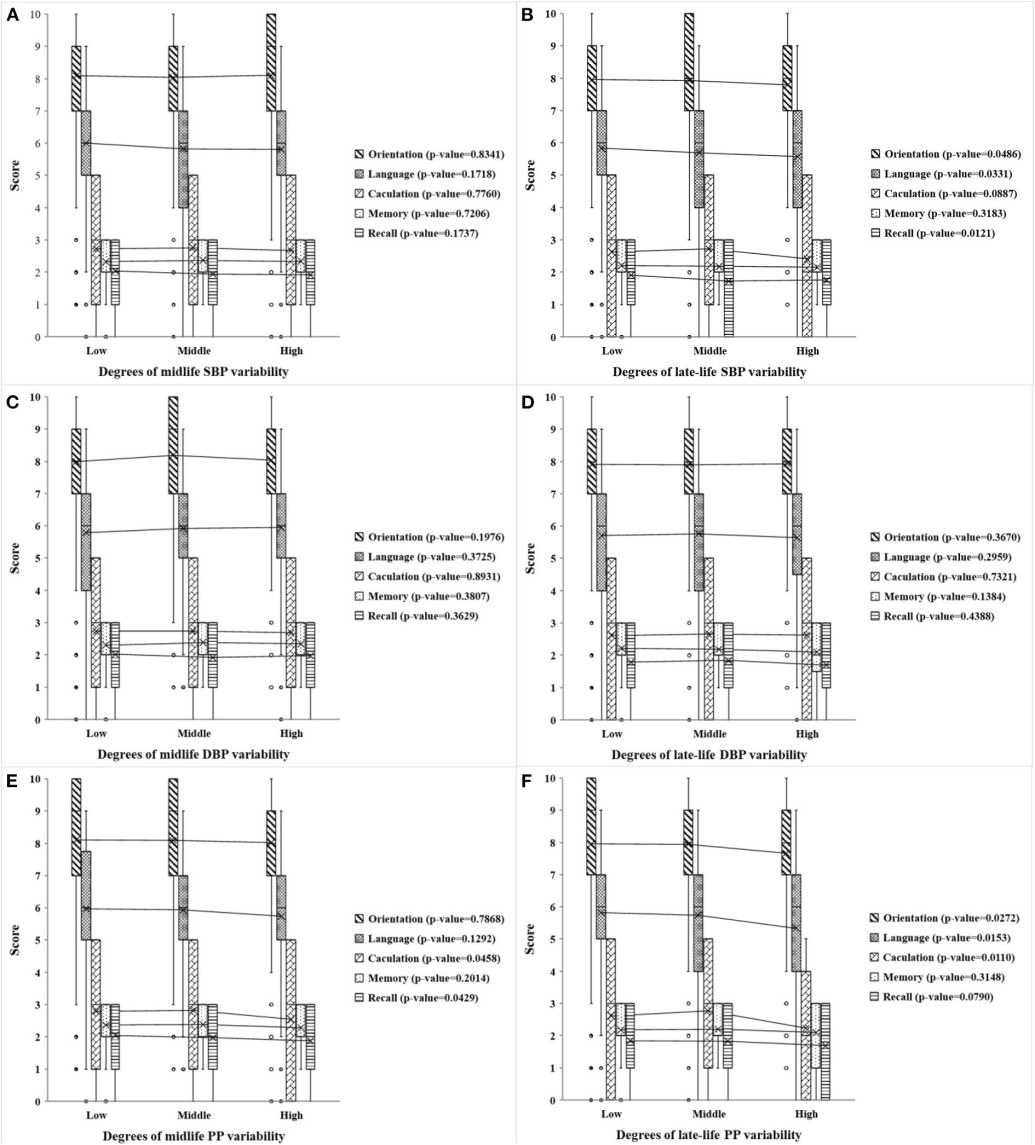
Scores of the five cognitive dimensions in different degrees of midlife and late-life visit-to-visit BP variability. **(A,B)** Respectively show the cognitive scores of five dimensions (including orientation, language, calculation, memory, and recall) in different degrees of midlife and late-life visit-to-visit SBP variability, **(C,D)** respectively, show the scores in different degrees of midlife and late-life visit-to-visit DBP variability, and **(E,F)** are the scores in different degrees of midlife and late-life visit-to-visit PP variability. The midlife visit-to-visit BP variability is present in the mid-old group, while the late-life visit-to-visit BP variability is present in the old-old group. Visit-to-visit SBP variability, DBP variability, and PP variability were divided by 3rd decile. Low, moderate and high represent the degree of variation. *P*-value is the analysis of variance of a certain cognitive dimension of different degrees of visit-to-visit BP variability.

Figure 3 shows the distribution and univariate analysis results of cognitive scores among participants with different degrees of midlife and late-life visit-to-visit BP variability. The data showed that participants with different degrees of late-life visit-to-visit SBP variability scored significantly differently while no significant difference was found in midlife visit-to-visit SBP variability. Low, middle, and high late-life visit-to-visit SBP variability groups differed significantly in cognitive scores of orientation, recall, language, and total (all *p*-value < 0.001) (*P*-value of total score were shown in Supplementary Table S2). However, no correlation was found between visit-to-visit DBP variability and cognitive scores of each dimension in both age groups. In addition, there were statistical differences among different degrees of midlife visit-to-visit PP variability in cognitive scores of calculation, recall, and total (all *p*-value < 0.05) (*P*-value of total score were shown in Supplementary Table S2), whereas significant differences were found in late-life visit-to-visit PP variability in cognitive scores of calculation, recall, language, and total (all *p*-value < 0.05) (*P*-value of total score were shown in Supplementary Table S2).

From the results summarized in Tables 2, 3, there were significantly negative associations between late-life visit-to-visit SBP variability and cognitive scores of orientation (adjusted β = −2.99, 95% CI −4.84 to −1.14, *p*-value = 0.002), recall (adjusted β = −1.69, 95% CI −2.89 to −0.48, *p*-value = 0.006), language (adjusted β = −2.26, 95% CI −4.13 to −0.38, *p*-value = 0.018), and total (adjusted β = −9.50, 95% CI −14.71 to −4.28, *p*-value < 0.001). However, midlife visit-to-visit SBP variability was only associated with language scores (adjusted β = −3.70, 95% CI −5.83 to −1.57, *p*-value = 0.001). This indicates that higher late-life visit-to-visit SBP variability was associated with worse cognitive performance, while weak relationship was found in the midlife visit-to-visit SBP variability and cognition.

Tables 2, 3 presented that midlife visit-to-visit DBP variability and PP variability showed a significant relationship with language score (adjusted β = 3.25, 95% CI −1.31 to −5.19 *p*-value = 0.001) and calculation score (adjusted β = −0.26, 95% CI −0.47 to −0.04 *p*-value = 0.022), respectively. However, no significant correlation was found between late-life visit-to-visit DBP variability, late-life PP variability, and cognitive score, which was inconsistent with the result of univariate analysis.

## Discussion

In this long-term follow-up study of the elderly adults, BP control status and visit-to-visit BP variability were associated with cognitive scores assessed by MMSE, analyzed by the fully adjusted general linear model. This study found that BP control status and visit-to-visit BP variability were significantly associated with cognitive function among the Chinese elderly, mainly in the dimension of orientation and language. Patients with late-life controlled hypertension scored higher than those with uncontrolled hypertension in language and those with untreated hypertension in orientation and language, and total. Of importance, visit-to-visit SBP variability showed great association with cognitive function of orientation, language, and recall. However, no significant correlation was found between midlife BP control status, late-life visit-to-visit DBP variability, late-life visit-to-visit PP variability, and cognitive score. This study contributes to highlight the potential role of antihypertensive therapy and keeping BP stable, especially SBP, in maintaining cognitive function of orientation and language and preventing dementia.

Our findings highlight the BP control status and visit-to-visit BP variability have different effects on different cognitive dimensions, suggesting that more attention should be paid to “orientation” and “language” in the elderly population. “Orientation” refers to “orientation to place” and “orientation to time”. “Orientation to place” reflects the function of position orientation, which was associated with toileting and getting lost (43), while “orientation to time” was related to basic living skills (including physical ambulation, dressing, grooming, and bathing) and daily medication management (26). Of note, patients with poor responsibility for medications tend to forget whether they have taken their medication or do not know when (43, 44). In China, 75.8% of the elderly suffered from at least one chronic disease (45), and it is vital to keep the elderly good orientation to time for disease management. Meanwhile, Studies showed that “language” [including “naming,” “repetition,” “3-stage command,” “writing,” and “copying” (41)] has relationships with various basic activities of daily living, including feeding, grooming, bathing, ability to make a phone call, shopping, food preparation, housekeeping, laundry, and ability to handle finances (26).

Our study contributes to clarify the association between BP control status and cognitive function of all dimensions, and emphasizes the importance of antihypertensive therapy in elderly patients with hypertension. Antihypertensive therapy may be associated with a slower rate of cognitive decline (46, 47), but the evidence is insufficient, plus few studies have focused on the ultimate control of blood pressure. We found that hypertensive patients with poor late-life BP control were more likely to get low cognitive scores, indicating that hypertensive patients should better control their blood pressure, which was consistent with Rouch et al.'s (13) and Ganguli et al.'s (48) results.

To better present the association between blood pressure and cognition, we divided the population into the mid-old and the old-old, and found the relationship between BP and cognition differed in the two age groups. In the general linear model, late-life BP control status showed significant relationships with cognitive function of orientation and language, while midlife BP control status showed no significant association with cognitive scores of all dimensions. In addition, midlife visit-to-visit DBP variability and PP variability were statistically correlated with cognition in language and calculation respectively, while these of late-life period showed no significant difference. In previous studies, few have compared the relationship between midlife/late-life BP and cognitive function, and the relationship between late-life BP and cognitive function in a large population. Therefore, this study contributes to comparing the relationship between midlife/late-life BP and cognitive function in the same population.

The significant relationship between late-life BP control status, visit-to-visit SBP variability and cognitive scores demonstrated that the elderly should pay more attention to cognition and receive effective antihypertensive treatment, which was similar to the results of Menezes's study (46). It might be related to both arterial increases and cognition decreases with aging (49–51). However, the associations between midlife/late-life BP and cognitive function in our analyses are inconsistent with previous studies (47). Previous studies indicated that no strong evidence showed that increased late-life BP was a risk factor for dementia (18), and midlife hypertension was associated with more cognitive decline (15). The differences between our findings and those of other studies may be due to factors such as race, definition of cognitive function, and duration of follow-up (16).

Our results indicated the inverse relationship between visit-to-visit SBP variability and cognitive scores, showing that keeping SBP stable is essential for maintaining good cognition, especially for orientation, recall, and language. Our analyses found that, after adjustment for all the covariates, the total MMSE score decreased by 9.50 units for every one-unit increase in the late-life visit-to-visit SBP variability, among which, orientation, recall, and language decreased by 2.99, 2.69, and 2.26 units, respectively. Besides, the language score decreased by 3.70 units for every one-unit increase in the midlife visit-to-visit SBP variability. Moreover, the correlation coefficients between visit-to-visit SBP variability and cognitive scores were significantly higher than those between BP control status and cognitive scores, indicating that reducing SBP fluctuation might be a key factor of both middle-aged and elderly adults to prevent dementia.

Unlike visit-to-visit SBP variability, late-life visit-to-visit DBP variability and visit-to-visit PP variability had no relationship with cognitive function according to the fully adjusted model. Also, the results of univariate analyses and general linear model regarding DBP and PP showed some differences, which may be because visit-to-visit BP variability was converted into a ranking variable in the univariates analyses, resulting in some loss of information. Further studies need to explore the effects of late-life visit-to-visit DBP and PP variability on cognitive function.

Current studies have demonstrated the relationship between visit-to-visit BP variability and cognition, and their results were partially consistent with this study. Nagai et al. (52) drew the same conclusion, demonstrating that MMSE score had significant negative correlations with the visit-to-visit SBP variability and DBP variability, and only the visit-to-visit SBP variability remained significantly different after confounders were adjusted. Alperovitch et al. (20) found that one standard deviation increase in visit-to-visit BP variability was associated with a 10% increase in dementia risk. However, most studies revealed the association between visit-to-visit BP variability and total cognition rather than the cognitive function of all dimensions, and our study noticed this and analyzed it in detail. In addition, there are some inconsistencies, so conclusions are still expected to be proved by more prospective studies.

Many studies have confirmed the physiological mechanism of the relationship between BP and cognitive function. One of the mechanisms that hypertension leads to cognitive impairment is inducing vascular alterations, which can lead to hypoperfusion, ischemic and hemorrhagic stroke, and white matter injury (16). One study reached similar conclusions, linking atherosclerosis and increased pressure pulsations with cerebrovascular injury and cognitive decline in middle-aged and elderly adults (53). Some studies have also confirmed that visit-to-visit BP variability is related to microvascular injury, endothelial damage, and vascular smooth muscle dysfunction (51, 52, 54, 55).

There were some limitations in this study. The indicators of demographic characteristics in this study relied on participants' self-report, such as living habits, antihypertensive treatment, and medical history. Despite the strict quality control measures taken by CHARLS, there may still be some reporting bias. Further, the cognitive function assessment was obtained from the latest survey, failing to carry out temporal analyses to reveal the cognitive change trend by years. Despite these limitations, the present study is the first long-term follow-up longitudinal research of Chinese people. Besides, this study focused on the relationship between BP control status at baseline, visit-to-visit BP variability, and cognitive scores in all cognitive dimensions, which is ignored by most studies.

## Conclusions

This study, based on a large elderly national data, reveals that the cognitive function of the Chinese elderly significantly correlated with BP control status at baseline and visit-to-visit BP variability. Our findings suggested that receiving effective late-life antihypertensive treatment might be of great importance to maintain good cognitive function and prevent dementia for elderly adults. Of importance, visit-to-visit SBP variability has the strongest correlation with cognitive function, indicating that the elderly should keep SBP stable. As our results show, more attention should be paid to the relationship between late-life BP control status, visit-to-visit BP variability, and cognitive function of orientation and language, which might contribute to prevent the development of cognitive impairment and dementia.

## Data availability statement

Publicly available datasets were analyzed in this study. This data can be found here: http://charls.pku.edu.cn/en/.

## Author contributions

Conceptualization and formal analysis: LX and DC. Methodology and writing—review and editing: LX, YY, and DC. Supervision: YY and DC. Writing—original draft: LX. All authors contributed to the article and approved the submitted version.

## Conflict of interest

The authors declare that the research was conducted in the absence of any commercial or financial relationships that could be construed as a potential conflict of interest.

## Publisher's note

All claims expressed in this article are solely those of the authors and do not necessarily represent those of their affiliated organizations, or those of the publisher, the editors and the reviewers. Any product that may be evaluated in this article, or claim that may be made by its manufacturer, is not guaranteed or endorsed by the publisher.
