# Supplementary material for: Association between blood pressure control status, visit-to-visit blood pressure variability, and cognitive function in elderly Chinese: A nationwide study
Source: Front Public Health. 2022 Aug 4;10:877192. doi: 10.3389/fpubh.2022.877192 (PMC9386068; doi:10.3389/fpubh.2022.877192)
Supplement: Supplementary file 1 [file Table_1.DOCX]

| Table S1. Cognitive scores of participants with different BP statuses at baseline (‾x±s) | | | | | | | | | | | | | | | | | | | | | | | |
| --- | --- | --- | --- | --- | --- | --- | --- | --- | --- | --- | --- | --- | --- | --- | --- | --- | --- | --- | --- | --- | --- | --- | --- |
| BP status at baseline | Orientation | | |  | Memory | | |  | Calculation | | |  | Recall | | |  | Language | | |  | Total | | |
|  | mid-old | old-old | Overall |  | mid-old | old-old | Overall |  | mid-old | old-old | Overall |  | mid-old | old-old | Overall |  | mid-old | old-old | Overall |  | mid-old | old-old | Overall |
| Controlled | 8.3±1.7 | 8.2±1.8 | 8.2±1.8 |  | 2.5±0.9 | 2.3±1.0 | 2.4±1.0 |  | 3.0±1.9 | 2.8±2.0 | 2.8±2.0 |  | 2.0±1.1 | 1.9±1.2 | 1.9±1.1 |  | 6.2±1.8 | 6.0±2.0 | 6.1±1.9 |  | 21.9±5.3 | 21.2±5.9 | 21.5±5.7 |
| Uncontrolled | 7.9±1.9 | 7.8±2.2 | 7.8±2.1 |  | 2.3±1.0 | 2.2±1.1 | 2.3±1.0 |  | 2.7±1.9 | 2.5±2.1 | 2.6±2.0 |  | 1.9±1.1 | 1.8±1.2 | 1.8±1.1 |  | 5.8±2.0 | 5.6±2.0 | 5.7±2.0 |  | 20.6±6.1 | 19.9±6.3 | 20.2±6.2 |
| Untreated | 8.1±1.9 | 7.5±2.2 | 7.8±2.1 |  | 2.3±1.0 | 2.1±1.1 | 2.2±1.0 |  | 2.5±2.0 | 2.4±2.0 | 2.5±2.0 |  | 1.9±1.1 | 1.8±1.2 | 1.8±1.1 |  | 5.9±2.0 | 5.4±2.1 | 5.6±2.1 |  | 20.8±5.9 | 19.2±6.4 | 19.8±6.3 |
| No | 8.1±1.9 | 7.9±2.0 | 8.0±1.9 |  | 2.4±1.0 | 2.2±1.1 | 2.3±1.0 |  | 2.8±2.0 | 2.7±2.0 | 2.7±2.0 |  | 2.0±1.1 | 1.8±1.2 | 1.9±1.1 |  | 5.9±2.0 | 5.7±2.0 | 5.8±2.0 |  | 21.0±5.8 | 20.3±6.1 | 20.6±5.9 |
| F | 1.12 | 5.64 | 4.94 |  | 0.71 | 2.47 | 3.14 |  | 1.44 | 2.37 | 3.90 |  | 0.51 | 0.63 | 0.43 |  | 1.49 | 4.14 | 4.70 |  | 1.38 | 5.11 | 6.17 |
| *p*-value | 0.3413 | 0.0008 | 0.0020 |  | 0.5459 | 0.0605 | 0.0244 |  | 0.2295 | 0.0685 | 0.0086 |  | 0.6727 | 0.5940 | 0.7322 |  | 0.2150 | 0.0061 | 0.0028 |  | 0.2486 | 0.0016 | 0.0004 |
