# Supplementary material for: Association between blood pressure control status, visit-to-visit blood pressure variability, and cognitive function in elderly Chinese: A nationwide study
Source: Front Public Health. 2022 Aug 4;10:877192. doi: 10.3389/fpubh.2022.877192 (PMC9386068; doi:10.3389/fpubh.2022.877192)
Supplement: Supplementary file 2 [file Table_2.DOCX]

Table S2. Cognitive score and cognitive impairment of participants with different degrees of BP variability

| BP variability | | Orientation | | |  | Memory | | |  | Calculation | | |  | Recall | | |  | Language | | |  | Total | | |
| --- | --- | --- | --- | --- | --- | --- | --- | --- | --- | --- | --- | --- | --- | --- | --- | --- | --- | --- | --- | --- | --- | --- | --- | --- |
|  |  | mid-old | old-old | Overall |  | mid-old | old-old | Overall |  | mid-old | old-old | Overall |  | mid-old | old-old | Overall |  | mid-old | old-old | Overall |  | mid-old | old-old | Overall |
| SBP variability | Low | 8.1±1.9 | 8.0±2.0 | 8.0±1.9 |  | 2.3±1.0 | 2.2±1.0 | 2.3±1.0 |  | 2.3±1.0 | 2.6±2.0 | 2.7±2.0 |  | 2.0±1.1 | 1.9±1.2 | 2.0±1.1 |  | 6.0±2.0 | 5.8±2.0 | 5.9±2.0 |  | 21.2±5.8 | 20.5±6.1 | 20.8±5.9 |
|  | Middle | 8.0±1.9 | 7.9±2.0 | 8.0±2.0 |  | 2.4±1.0 | 2.2±1.1 | 2.3±1.0 |  | 2.4±1.0 | 2.7±2.0 | 2.7±2.0 |  | 1.9±1.1 | 1.7±1.2 | 1.8±1.2 |  | 5.8±2.0 | 5.7±2.1 | 5.8±2.1 |  | 20.9±5.9 | 20.3±6.2 | 20.6±6.1 |
|  | High | 8.1±1.9 | 7.7±2.1 | 7.9±2.0 |  | 2.3±0.9 | 2.1±1.1 | 2.2±1.0 |  | 2.3±0.9 | 2.5±2.0 | 2.6±2.0 |  | 1.9±1.1 | 1.8±1.2 | 1.8±1.1 |  | 5.8±1.8 | 5.5±2.0 | 5.6±2.0 |  | 20.8±5.6 | 19.6±6.2 | 20.1±6.0 |
| F | | 0.18 | 3.03 | 1.85 |  | 0.33 | 1.15 | 1.12 |  | 0.25 | 2.43 | 2.41 |  | 1.75 | 4.42 | 6.66 |  | 1.76 | 3.42 | 5.37 |  | 0.49 | 3.92 | 4.43 |
| p-value | | 0.8341 | 0.0486 | 0.1578 |  | 0.7206 | 0.3183 | 0.3268 |  | 0.7760 | 0.0887 | 0.0900 |  | 0.1737 | 0.0121 | 0.0013 |  | 0.1718 | 0.0331 | 0.0047 |  | 0.6124 | 0.0199 | 0.0119 |
| DBP variability | Low | 8.0±1.9 | 7.9±2.0 | 7.9±2.0 |  | 2.3±1.0 | 2.2±1.0 | 2.3±1.0 |  | 2.7±2.0 | 2.6±2.0 | 2.7±2.0 |  | 2.0±1.1 | 1.8±1.2 | 1.9±1.1 |  | 5.8±2.1 | 5.7±2.0 | 5.7±2.1 |  | 20.8±6.1 | 20.2±6.1 | 20.5±6.1 |
|  | Middle | 8.2±1.9 | 7.9±2.0 | 8.0±2.0 |  | 2.4±0.9 | 2.2±1.0 | 2.3±1.0 |  | 2.7±1.9 | 2.7±2.0 | 2.7±2.0 |  | 1.9±1.1 | 1.8±1.2 | 1.9±1.1 |  | 5.9±1.9 | 5.8±2.1 | 5.8±2.0 |  | 21.2±5.6 | 20.3±6.3 | 20.7±6.0 |
|  | High | 8.0±1.9 | 7.8±2.1 | 7.9±2.0 |  | 2.3±1.0 | 2.1±1.1 | 2.2±1.0 |  | 2.7±1.9 | 2.6±2.0 | 2.6±2.0 |  | 2.0±1.1 | 1.8±1.2 | 1.9±1.1 |  | 5.9±1.9 | 5.6±2.0 | 5.7±1.9 |  | 21.0±5.7 | 19.8±6.2 | 20.3±6.0 |
| F | | 1.62 | 1.00 | 1.56 |  | 0.97 | 1.98 | 1.36 |  | 0.11 | 0.31 | 0.40 |  | 1.01 | 0.82 | 0.36 |  | 0.99 | 1.22 | 0.75 |  | 0.42 | 1.49 | 1.24 |
| p-value | | 0.1976 | 0.3670 | 0.2108 |  | 0.3807 | 0.1384 | 0.2555 |  | 0.8931 | 0.7321 | 0.6695 |  | 0.3629 | 0.4388 | 0.6964 |  | 0.3725 | 0.2959 | 0.4719 |  | 0.6562 | 0.2257 | 0.2908 |
| PP variability | Low | 8.1±1.9 | 8.0±2.1 | 8.0±2.0 |  | 2.4±1.0 | 2.2±1.1 | 2.3±1.0 |  | 2.8±1.9 | 2.6±2.0 | 2.7±2.0 |  | 2.0±1.1 | 1.8±1.2 | 1.9±1.1 |  | 6.0±2.1 | 5.8±2.0 | 5.9±2.1 |  | 21.3±5.9 | 20.4±6.2 | 20.8±6.1 |
|  | Middle | 8.1±1.9 | 7.9±1.9 | 8.0±1.9 |  | 2.4±0.9 | 2.2±1.0 | 2.3±1.0 |  | 2.8±2.0 | 2.8±2.0 | 2.8±2.0 |  | 2.0±1.1 | 1.8±1.2 | 1.9±1.1 |  | 5.9±1.9 | 5.7±2.1 | 5.8±2.0 |  | 21.2±5.7 | 20.5±6.0 | 20.8±5.9 |
|  | High | 8.0±1.9 | 7.7±2.1 | 7.8±2.0 |  | 2.3±1.0 | 2.1±1.1 | 2.2±1.0 |  | 2.5±2.0 | 2.4±2.0 | 2.5±2.0 |  | 1.9±1.1 | 1.7±1.2 | 1.8±1.2 |  | 5.7±1.8 | 5.5±2.0 | 5.6±1.9 |  | 20.5±5.8 | 19.5±6.3 | 19.9±6.1 |
| F | | 0.24 | 3.61 | 3.52 |  | 1.60 | 1.16 | 3.06 |  | 3.09 | 4.52 | 7.52 |  | 3.15 | 2.54 | 6.12 |  | 2.05 | 4.19 | 6.64 |  | 3.06 | 5.67 | 9.40 |
| p-value | | 0.7868 | 0.0272 | 0.0296 |  | 0.2014 | 0.3148 | 0.0469 |  | 0.0458 | 0.0110 | 0.0006 |  | 0.0429 | 0.0790 | 0.0022 |  | 0.1292 | 0.0153 | 0.0013 |  | 0.0472 | 0.0035 | 0.0001 |

SBP variability, DBP variability, and PP variability were divided by 3rd decile. Low, moderate and high represent the degree of variation.
